# Subliminal priming modulates motor sequence learning

**DOI:** 10.3758/s13421-024-01668-8

**Published:** 2024-11-21

**Authors:** Michael William Simpson, Jing Wu, Zheng Ye

**Affiliations:** 1 Institute of Neuroscience, Center for Excellence in Brain Science and Intelligence Technology, Chinese Academy of Sciences, Shanghai, 200031 China; 2 University of Chinese Academy of Sciences, Beijing, 100049 China

**Keywords:** Subliminal masked prime, Motor sequence learning, Selection, Inhibition, Conflict

## Abstract

Sequential behaviour is underpinned by the selection and inhibition of movement at appropriate points in space and time. Sequences embedded among movement patterns must be learnt, yet the contribution of response selection and inhibition to the acquisition of motor sequences remains poorly understood. We addressed this issue by overlaying the serial reaction time task (SRTT) with subliminal masked primes that differentially weighed response tendencies. In Experiment 1, twenty-four healthy young adults, and in Experiment 2, thirty-six participants, performed the SRTT with congruent (same position), incongruent (different position), or neutral (no prime) subliminal masked primes. Each condition featured an embedded eight-digit (Experiment 1) or ten-digit (Experiment 2) second-order sequence, with conditions presented in counterbalanced order during a single session. Sequence specific learning was observed under neutral and congruent prime conditions. Independent of sequence awareness, congruent primes reduced initial response latency and led to greater sequence specific learning compared with neutral primes. However, incongruent primes appeared to attenuate learning (Experiment 1). These results demonstrate that prime congruency modulates sequence specific learning below the threshold of conscious awareness. Congruent primes may elevate the salience of stimulus–response compounds and accentuate learning, but at the cost of increased awareness. Incongruent primes, and the induction of response conflict, attenuate sequence specific learning (Experiment 1) and may prevent the formation of cross-temporal contingencies necessary for implicit motor sequence learning.

**Supplementary information:**

The online version contains supplementary material available at 10.3758/s13421-024-01668-8.

## Introduction

Procedural memory and the implicit formation of higher-order associations support temporally organised behaviour and allow for the prediction of future events (Keele et al., 2003; Robertson, 2007). This form of learning (i.e., implicit) is thought to underpin complex sequence-based behaviour in which movements are selected and inhibited at appropriate points in space and time (Horváth et al., 2022). However, the contribution of response selection and inhibition (i.e., response control) to the implicit acquisition of sequential motor behaviour remains poorly understood, an obscurity that may be tapped by priming response tendencies.

The study of implicit learning has extensively employed variations of the serial reaction time task (SRTT), in which a response is made to one of several targets over blocks that follow a predetermined sequence (Nissen & Bullemer, 1987). Response times typically decrease as participants benefit from implicit knowledge of the sequence but increase with the introduction of random trials. The difference in response times between sequence and random trials is thought to reveal sequence specific learning (Robertson, 2007). The knowledge that underpins this learning effect is embedded in complex associations between stimuli and responses, the locus of which remains debated among three primary hypotheses. Stimulus-based theory posits that associations are learnt between stimuli during stimulus encoding (Clegg, 2005), whereas response-based theory implies that associations are drawn between responses during response execution (Bischoff-Grethe et al., 2004). On the other hand, stimulus–response-based theory suggests that associations are formed between stimuli and responses during response selection (Schumacher & Schwarb, 2009). Irrespective of conjecture, the formation of cross-temporal contingencies and associations among stimulus and/or response compounds are shaped by factors including attention (Song, 2019) and feedback (Shea & Wulf, 1999).

The congruency of information that guides sequential behaviour has a profound effect not only on performance but on the rate at which we adapt and learn sequential behaviour. Incongruent online feedback, for example, can taper performance gains during a sequential finger tapping task (Ossmy & Mukamel, 2018), whereas modifying the stimulus–response map of the SRTT has been shown to induce steeper performance gains and larger sequence specific learning (Deroost & Soetens, 2006; Koch, 2007). Stimulus–response remapping demands that conventional one-to-one responses are suppressed to elicit an appropriate response, indicating a role for altered response control during the acquisition of sequential motor skill. Indeed, overt suppression of sequential motor memory is known to attenuate task consolidation (Schmidt et al., 2023), a process thought to be mediated by increased beta oscillatory power (Tempel et al., 2020). Moreover, the costs associated with serial task switching (i.e., the backward inhibition effect) illustrate how serial behaviour is subject to inhibitory control (Mayr & Keele, 2000). Despite the association between states of response control and the production of serial behaviour (Fales et al., 2006; Schmidt et al., 2023; Takacs & Beste, 2023), it remains unclear how information processed beyond our awareness mediates the acquisition of sequential motor skill. Implicit learning, driven by low-level automatic processes that operate outside of conscious awareness, typically bypasses higher-order executive functions. Yet attempts to modulate demands on response control have relied on response remapping (Deroost & Soetens, 2006), dual-task (Schumacher & Schwarb, 2009), or distractor methodology (Takacs & Beste, 2023) and fail to examine response control during sequence learning without evoking additional cognitive resources (i.e., executive attention). A modulation of implicit learning by lower-level mechanisms of response control would signal the independence of basic learning mechanisms from conscious awareness and reveal the boundaries of implicit learning, a challenge that can be addressed by subliminally priming response tendencies.

Subliminal priming, the provision of a stimulus prior to a response, can modulate the speed at which an upcoming response is generated (Taylor & McCloskey, 1990). When shown briefly (<50 ms) and followed by a mask, priming permits a window through which the motor system can be manipulated beyond awareness and allows for the differential weighing of response tendencies to examine behavioural control under conflict (Kiesel et al., 2007). Moreover, response priming permits learning phenomena and response bias manipulations to co-occur in the same conceptual area (i.e., implicit) and minimises the engagement of secondary cognitive and motor resources. Learning-related changes in sequential motor performance are driven by the optimisation of motor planning in which movement details are specified beyond stimulus identification (Ariani & Diedrichsen, 2019). Priming responses allows us to directly tap this locus of sequential behaviour and either facilitate (congruent prime) or attenuate (incongruent prime) response selection by modulating the congruency between the primed and actual stimuli.

**Fig. 1:**
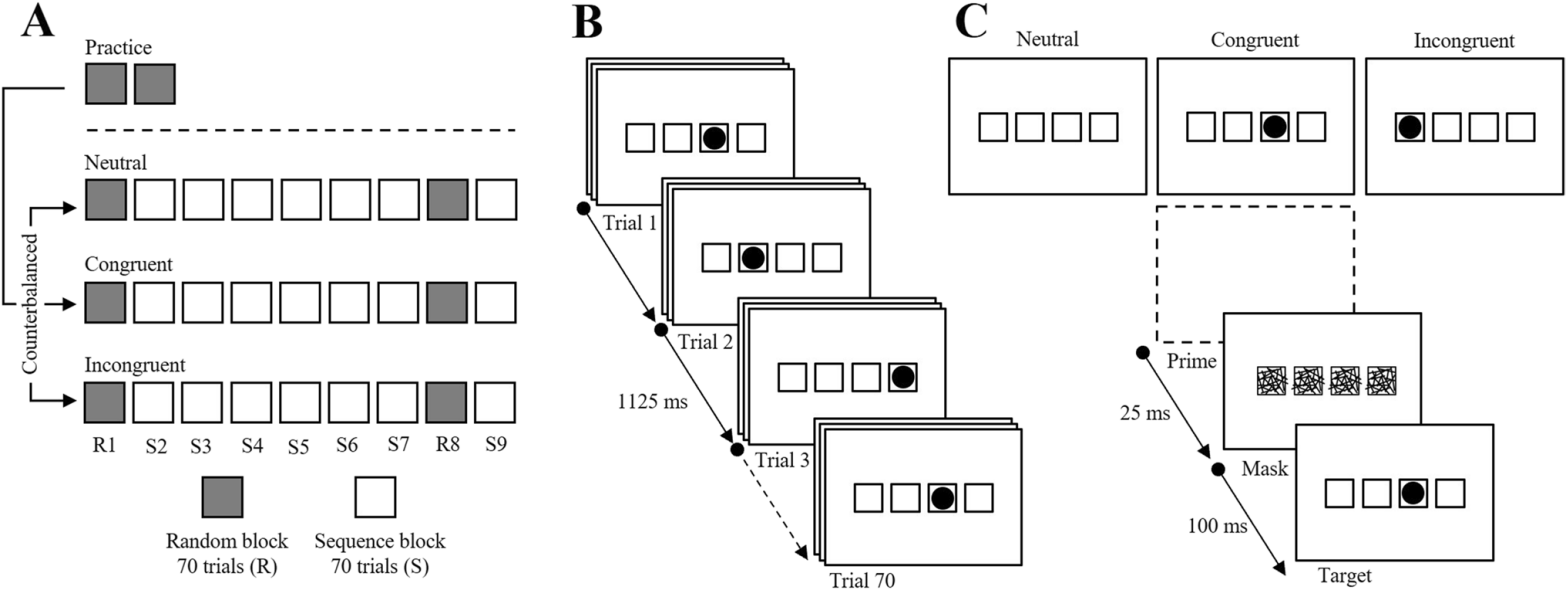
Experiment 1 design (**A**) and SRTT (**B**) overlaid with subliminal masked primes (**C**)

To investigate the role of implicit response control in implicit motor sequence learning, we conducted two experiments in which the SRTT was overlaid with subliminal masked primes. These primes were either congruent (same position) or incongruent (different position) relative to the upcoming target, and response times were compared with performance under neutral conditions (no prime; see Fig. 1). We hypothesised that congruent primes would bias response tendencies toward the target and augment learning by reducing response conflict and strengthening stimulus–response associations. Conversely, incongruent primes would bias responses toward incorrect targets, necessitating greater inhibitory control and potentially disrupting the formation of cross-temporal associations.

## Experiment 1

### Methods

The aim of Experiment 1 was to explore the effect of subliminal response priming on implicit motor sequence learning during the SRTT.

#### Participants

Stable learning effects using the SRTT have been observed with samples of 21 or more participants (Stark-Inbar et al., 2016). Accounting for a 10% loss in data, 24 healthy young university students were recruited for Experiment 1 (12 women; 25.1 ± 2.5 years, mean ± *SD*). Inclusion criteria were 18 to 40 years of age, right-handed, free from neurologic or psychiatric disorder, cognitive impairment (Montreal Cognitive Assessment ≥ 26/30) and depression (Beck Depression Inventory–Short Form ≤ 9; Furlanetto et al., 2005). This study was approved by the ethics committee of the Center for Excellence in Brain Science and Intelligence Technology in accordance with the Declaration of Helsinki.

#### Experimental design and procedure

Participants attended one experimental session in which baseline corticospinal excitability was measured using transcranial magnetic stimulation (TMS) before repeating the SRTT under three conditions: neutral, congruent primes, and incongruent primes. For all TMS-related methodology and results, please see Supplement One. To minimise potential order effects, a Latin square counterbalanced design was applied. Following the SRTT, participants completed a brief sequence and prime awareness questionnaire. Experimental sessions lasted 70–90 min.

#### Serial reaction time task and masked primes

The SRTT was administered using Presentation software (Version 23.1; Neurobehavioral Systems Inc., USA) and displayed on a computer monitor at 120 Hz (ASUS, VG256QM). Each trial consisted of a black circle (26 mm ø, 2.4°) presented in one of four horizontally aligned black squares (34 mm^2^, 3.2°; separated by gaps of 9 mm, 0.8°) on a white background. Participants were instructed to press the button on a computer keyboard corresponding to the location of the black circle as quickly and accurately as possible. Participants used their right hand by pressing the ‘c’, ‘v’, ‘b’, and ‘n’ keys with their index, middle, ring, and little fingers, respectively.

Participants first completed two practice blocks (70 trials each) under neutral prime conditions before performing the SRTT three times. The SRTT consisted of nine experimental blocks, each containing 70 trials, and lasted exactly 1 min and 18 s. At the beginning of each SRTT a fixation mark (‘+’) was displayed followed by the first task block. Each trial featured a target presented for a maximum of 1,000 ms. If a response was registered within this time, the target would disappear, and the remaining time would play out before the next stimulus appeared. If no response was made, the target remained on-screen until the time elapsed. Response times and errors were recorded for each trial, and no feedback was provided throughout the experiment. In Blocks 1 and 8, target presentation was pseudorandomised (random blocks), while Blocks 2–7 and Block 9 followed a second-order eight-digit sequence (sequence blocks). Each experimental condition featured a different sequence: neutral (1-3-2-4-3-1-4-2), congruent (2-3-1-3-4-2-4-1), and incongruent (3-4-2-1-2-4-3-1). Among sequenced blocks, the starting point of each sequence varied; the first sequenced block began at Position 1, while subsequent sequence blocks started two positions back.

Prior to each target stimulus, a prime was displayed for 25 ms, followed by a mask for 100 ms. The prime, identical to the target stimulus, appeared in either a congruent or incongruent position (see Fig. 1). Masks consisted of 20 lines of random length and orientation within each of the four horizontally aligned squares. During each interblock interval (15 s), a message informed participants of the break and the upcoming block. Participants received a 5-min break between each SRTT. After completing all three SRTTs, participants were asked whether they noticed a repeating sequence and to type or verbalise their observations. Participants were then asked if they noticed any shape presented before the mask, and if yes, were encouraged to draw it, allowing for nondirected quantification of prime awareness.

#### Data processing and statistical analysis

Data were imported into MATLAB (2020b, The MathWorks, USA), where erroneous responses (incorrect and missed trials) were removed before calculating median response times for each block. The cleaned data were then imported into SPSS (Version 26, IBM, USA). A two-way repeated-measures analysis of variance (ANOVA) with factors Block (R1, R8) and Prime (neutral, congruent, incongruent) examined changes in baseline motor function across conditions. To verify the baseline effect of our experimental manipulation, a one-way between-subjects ANOVA compared response times between Practice Block 2 and the first block of the first condition (R1). Using a Latin square counterbalancing method, eight participants experienced neutral, congruent, and incongruent conditions as their first respective condition. To test for differences in sequence-specific learning among conditions, response times from sequence Blocks 7 and 9 were combined and compared with those from random Block 8 using a two-way repeated-measures ANOVA with factors Sequence (sequence, random) and Prime. Changes in response latency across sequenced blocks (S2 through S7) were analysed using a two-way repeated-measures ANOVA with factors Block (S2, S3, S4, S5, S6, S7) and Prime. Post hoc one-way ANOVAs and pairwise *t* tests (corrected for multiple comparisons using Bonferroni) were employed to examine significant interactions and main effects.

All data were assessed for normality using the Shapiro–Wilk test, and data that did not conform to a normal distribution were analysed using nonparametric equivalent tests. The Greenhouse–Geisser correction factor was applied when assumptions of homogeneity of variance were violated. An alpha level of 0.05 was set to denote statistical significance. All values are presented as mean ± standard error unless otherwise stated.

#### Data availability

Data that support the findings of this study can be found on Science Data Bank (10.57760/sciencedb.12986).

### Results

Twenty-four participants completed Experiment 1. Six participants were aware of and could recall ≥50% of one or more sequences and were removed from the primary analysis. Notably, the sequence recalled by all six participants was presented in the congruent prime condition. One further participant was removed due to responses that exceeded three times the standard deviation of the group mean in Blocks 7 and 8. Of the 17 participants included in the final analysis, five reported sequence awareness without sequence recall (see Table 1). Erroneous responses peaked at 2.35 ± 0.56 errors per block (<2%) and remained stable across prime conditions with very few missed trials (<0.2%).

**Table 1:** Demographic information and sequence awareness

	Gender (M/W)	Age	MoCA	BDI-SF
**Experiment **1	12/12	25 ± 3	29 ± 1	2 ± 2
Unaware	5/7	25 ± 2	29 ± 1	3 ± 2
Aware, no recall	4/2	23 ± 2	30 ± 1	1 ± 1
Aware, with recall	3/3	27 ± 3	29 ± 1	2 ± 1
**Experiment **2	15/13	24 ± 3	29 ± 1	3 ± 2

Mean ± standard deviation. Montreal Cognitive Assessment, MoCA; Beck Depression Inventory Short Form, BDI-SF

#### Effect of prime congruency on response latency

Among random blocks (R1, R8), response latency shifted according to prime congruency (prime main effect: *F*_2,32_ = 26.65, *p* < .001, η_p_^2^ = 0.63, β = 1.00), yet remained stable from the first to the second random block (block main effect: *F*_1,16_ = 0.89, *p* = .36, η_p_^2^ = 0.05, β = 0.14) and among prime conditions (prime-by-block interaction: *F*_1.4,22.5_ = 1.77, *p* = .20, η_p_^2^ = 0.10, β = 0.28). Compared with response latency under neutral prime conditions, responses were significantly faster under congruent (−57.65 ± 11.08 ms; *p* < .001), but not incongruent primes (6.56 ± 9.03 ms; *p* = 1.00). The shift of response latency was further confirmed by comparing response times between the second practice block and the first random block of the first respective condition. Note that all 24 participants were included in this analysis given the independence of sequence awareness at this stage of the experiment. Response times during practice were comparable among groups that performed the SRTT under neutral, congruent, or incongruent primes as their first respective condition (*F*_2,21_ = 1.81, *p* = .19, η_p_^2^ = 0.15, β = 0.33). Yet only response times under congruent prime conditions were significantly faster than practice (−49.16 ± 28.01 ms; *p* = .005), indicating that a prime mediated shift in response latency was present at baseline and not a consequence of practice or task exposure.

#### Effect of prime congruency on sequence specific learning

**Fig. 2:**
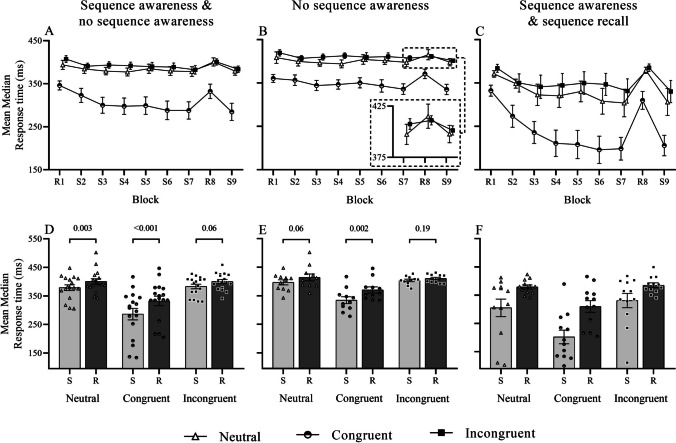
Mean median response times and standard error of a SRTT primed with neutral, congruent, or incongruent targets (**A, B, C**) and sequence specific learning evaluated over sequenced (S7, S9) and random (R8) blocks **(D, E, F)** among individuals with and without sequence awareness and or recall

Response latency in blocks seven through nine differed among prime conditions (prime main effect: *F*_1.3,21.1_ = 31.79, *p* < .001, η_p_^2^ = 0.67, β = 1.00). Responses were significantly faster under congruent (−80.3 ± 14.0 ms; *p* < .001) but not incongruent primes (2.06 ± 6.27 ms; *p* = 1.00) when compared with neutral. Sequence specific learning was broadly observed (sequence main effect: *F*_1,16_ = 25.01, *p* < .001, η_p_^2^ = 0.61, β = 0.99) in that response times of sequenced blocks (S7, S9) were significantly faster than random blocks (R8) (−28.6 ± 5.7 ms; *p* < .001). Moreover, sequence specific learning differed among the three prime conditions (prime-by-sequence interaction: *F*_2,32_ = 7.64, *p* = .002, η_p_^2^ = 0.32, β = 0.93). Response times among sequenced blocks were significantly faster than random blocks under both neutral (*t*_16_ = −3.76, *p* = .003, *d* = −0.91; one-tailed) and congruent primes (*t*_16_ = −5.63, *p* < .001, *d* = −1.37), but just missed significance under incongruent primes (*t*_16_ = −2.24, *p* = .06, *d* = −0.54; Fig. 2A, D). When compared with neutral, the difference in response times under sequenced and random blocks was significantly larger under congruent (*t*_16_ = 3.40, *p* = .007, *d* = −0.79) but not incongruent primes (*t*_16_ = −0.45, *p* = 1.00, *d* = 0.14).

#### Rate of performance gain

Compared with baseline, response times decreased significantly from −15.9 ± 3.5 at S1 to −32.7 ± 8.0 at S7 (block main effect: *F*_2.4,38.5_ = 4.01, *p* = .02, η_p_^2^ = 0.20, β = 0.74). However, the change of response times differed among the three prime conditions (prime main effect: *F*_2,32_ = 5.60, *p* = .008, η_p_^2^ = 0.26, β = 0.82), with a greater decrease of response times observed under congruent (−33.82 ± 12.03 ms) but not incongruent primes (3.99 ± 9.77 ms) when compared with neutral. Moreover, the rate of change was not equal (prime-by-block interaction: *F*_10,160_ = 2.52, *p* = .008, η_p_^2^ = 0.14, β = 0.94). Differences between prime conditions emerged from block S3 onwards (*F*_2,32_ = 5.03, *p* = .01, η_p_^2^ = 0.24, β = 0.78) where response times under congruent (−31.0 ± 12.1 ms, *p* = .06), but not incongruent primes (−1.89 ± 10.33 ms, *p* = 1.00), decreased more than neutral, a trend that broadly continued through to block S7 (Fig. 2A).

#### Influence of sequence awareness on sequence specific learning

To evaluate potential bias stemming from sequence awareness, we repeated the analyses among participants with no sequence awareness. Note, one additional participant was removed from this analysis due to outlying response times under incongruent primes. Data from 11 participants with no sequence awareness were carried forward. Prime congruency and sequence specific learning effects were again observed (prime main effect: *F*_2,20_ = 14.29, *p* < .001, η_p_^2^ = 0.59, β = 1.00; sequence main effect: *F*_1,10_ = 15.24, *p* = .003, η_p_^2^ = 0.60, β = 0.94), with the degree of learning unequal among conditions (prime-by-sequence interaction: *F*_2,20_ = 8.03, *p* = .003, η_p_^2^ = 0.45, β = 0.92). Pairwise comparisons confirmed sequence specific learning under congruent prime conditions (*t*_10_ = −4.57, *p* = .002, *d* = −1.38; one-tailed) and showed a trend toward significance under neutral primes (*t*_10_ = −2.33, *p* = .06, *d* = −0.70), but no significant effect was found under incongruent primes (*t*_10_ = −.66,* p* = .19, *d* = −0.50; Fig. 2B, E). Despite a lack of sequence specific learning under incongruent prime conditions, the difference in response times between sequence and random blocks was comparable to neutral (*t*_10_ = −1.60, *p* = .28, *d* = −0.01). In contrast, sequence specific learning under congruent primes was considerably larger than under neutral primes (*t*_10_ = −2.45, *p* = 0.07, *d* = −0.81). Non-normal distribution of response time data among individuals with sequence awareness and/or sequence recall prevented multilevel comparisons of awareness levels on primed sequence learning. However, with sequence awareness/recall, the change of response times from sequence to random blocks was comparable among prime conditions, χ^2^_(2)_ = −4.6, *p* = .10.

## Experiment 2

We identified in Experiment 1 that the presentation of subliminally masked primes prior to a stimulus could modify sequence-specific learning in the SRTT. Although well powered, observations among individuals without sequence awareness were made with only 11 participants. Furthermore, we were unable to determine if (1) sequence awareness remained stable throughout the experimental session and (2) the degree to which participants were aware of the masked primes. To address these concerns and further validate our previous observations, we conducted a second experiment deploying a ten-digit second-order sequence to help mitigate the development of sequence awareness. Additionally, a choice task was introduced to evaluate sequence awareness after each condition, and a mask test to determine individual prime identification thresholds. By incorporating the choice task and mask test, we enhance our confidence that both the observations and manipulations in our experiment are occurring below the threshold of awareness, ensuring that subliminal prime manipulation and implicit learning are effectively aligned. The methodology of Experiment 2 closely replicates that of Experiment 1, with the key modifications outlined below.

### Methods

#### Participants

Thirty-six healthy young university students (13 women; 24 ± 3 years, mean ± *SD*) were recruited for Experiment 2. Sample size was determined using G*Power (Version 3.1.9.7; University of Kiel, Germany) according to the effect sizes (partial eta-squared; η_p_^2^) observed in Experiment 1. To achieve a significant interaction effect (*p* = .05, β = 0.95) using a two-way repeated-measures ANOVA assuming an effect size between 0.32-0.45 (see Experiment 1 results), a sample of 27–39 participants would be required. To allow for effective counterbalancing, 36 participants were recruited. This study was approved by the ethics committee of the Center for Excellence in Brain Science and Intelligence Technology in accordance with the Declaration of Helsinki.

#### Experimental design and procedure

Participants attended one experimental session and performed the SRTT under three conditions: neutral, congruent primes, and incongruent primes. Following each SRTT, participants performed a choice task. At the end of the session, participants completed a brief sequence and prime awareness questionnaire followed by a mask test. Experimental sessions lasted 70–80 min.

#### Serial reaction time task and masked primes

Two practice sessions, each comprising three blocks of 20 trials under neutral, congruent, and incongruent primes, were conducted before participants repeated the SRTT three times. Each SRTT consisted of seven blocks of 80 trials, with each block lasting exactly 1 min and 28 s. Blocks 2–5 and block 7 followed a ten-digit second-order sequence (1-2-4-1-3-2-1-4-3-4; 2-4-3-1-4-2-3-2-1-3; 3-1-2-3-2-4-1-3-4-2), while blocks 1 and 6 followed a random sequence. Condition order and sequence were counterbalanced among participants using a dual Latin square design. Before each target stimulus, a prime was presented for 16.7 ms followed by a mask for 83.3 ms. Prime duration was reduced to help mitigate the development of prime awareness (Schräder et al., 2023). Each block was separated by an interblock interval of 18–20 s (randomly jittered), during which the mean reaction time of the previous block was displayed on-screen. All stimuli were presented against a middle-grey background (RGB; 124, 124, 124).

#### Choice task

Given that explicit knowledge can develop with increasing incidental task practice (Gaschler et al., 2014), a choice task consisting of two blocks of 30 trials was conducted after each SRTT to assess explicit sequence awareness (Haider et al., 2011). Block 1 followed a random sequence, while block 2 adhered to the second-order sequence from the previous SRTT. In ten pseudorandom trials, separated by a minimum of two trials, all four target positions were filled in black to conceal the target location. Participants were instructed that the target was hidden under one of the boxes and asked to select the position they believed the target would appear. Each choice trial lasted a maximum of 5,000 ms. In the sequence block, each element of the ten-digit sequence was tested as a choice trial. In the random block, an equal representation of the ten-digit sequence response locations was tested. We hypothesised that making response selection an explicit process, sequence awareness pertaining to second-order stimulus transitions would lead to greater response accuracy compared with random stimuli.

#### Mask test

A mask test was conducted at the end of the experiment to evaluate the effectiveness of prime masking. This test consisted of one block of 100 trials, presented under both congruent and incongruent SRTT conditions. Stimuli followed a random sequence and were preceded by either a congruent or incongruent prime, with prime congruency randomised and equally represented (50% congruent, 50% incongruent). Participants were informed that the prime would appear before the target but were instructed to respond only to the prime’s location. If they could not identify the prime location, they were to respond spontaneously. The first trial began with the presentation of an unmasked prime. The number of lines forming the mask was adjusted on a trial-by-trial basis using a fixed-step, one-up/two-down procedure: one line was added to the mask after each correct response and two lines removed for each incorrect response. This method aimed for correct prime identification to converge at 66% (Kaernbach, 1991).

#### Data processing and statistical analysis

The first three responses from each block were removed due to spuriously long response times. To test for differences in sequence-specific learning among conditions, response times from sequence blocks 5 and 7 were combined and compared with those from random block six. A two-way repeated-measures ANOVA was used to evaluate differences in choice task accuracy among sequence and random blocks across prime conditions.

### Results

Thirty-six participants completed Experiment 2. Eight participants were removed from data analysis: four due to sequence awareness and recall (≥50% of one or more sequences), one due to explicit prime awareness, and three due to fatigue or inattentiveness, as indicated by spurious increases in response times from random block 1 to sequence block 5. Of the remaining 28 participants, 14 reported the use of a response strategy pertaining to four-digit chunks (i.e., if the previous three response locations were 1-3-2, the next response should be location 4). Given the absence of structured knowledge pertaining to a ten-digit sequence, these participants were retained, and data from all 28 participants were carried forward for analysis. Response error peaked at 5.6 ± 0.7 errors per block (7%) and remained stable across prime conditions with no missed trials. See Table 1 for demographic information.

#### Effect of prime congruency on response latency

Response latency was significantly different among prime conditions (prime main effect: *F*_2,54_ = 53.5, *p* < .001, η_p_^2^ = 0.63, β = 1.00), and in contrast to Experiment 1, changed over the course of the task (block main effect: *F*_1,27_ = 10.46, *p* = .003, η_p_^2^ = 0.28, β = 0.88), a change that was not equal among prime conditions (prime-by-block interaction: *F*_1.2,23.2_ = 7.92, *p* = .005, η_p_^2^ = 0.23, β = 0.83). Compared with neutral prime conditions, response latency was significantly faster under congruent primes (−32.7± 6.80 ms, *p* < .001) and slightly slower under incongruent primes (15.59 ± 7.21, *p* = .08). Response latency decreased significantly by block 6, but only under congruent prime conditions (*t*_27_ = −3.21, *p* = 0.01, *d* = −0.61). The shift of response latency was again confirmed by comparing response times from the second round of practice among 35 participants (one excluded due to log file error; *F*_2,68_ = 43.25, *p* < .001, η_p_^2^ = 0.56, β = 1.00). Response times were significantly faster under congruent (−37.75 ± 5.04 ms; *p* ≤ .001), but not incongruent primes (1.47 ± 4.73 ms; *p* = 1.00), when compared with neutral.

#### Effect of prime congruency on sequence specific learning

**Fig. 3:**
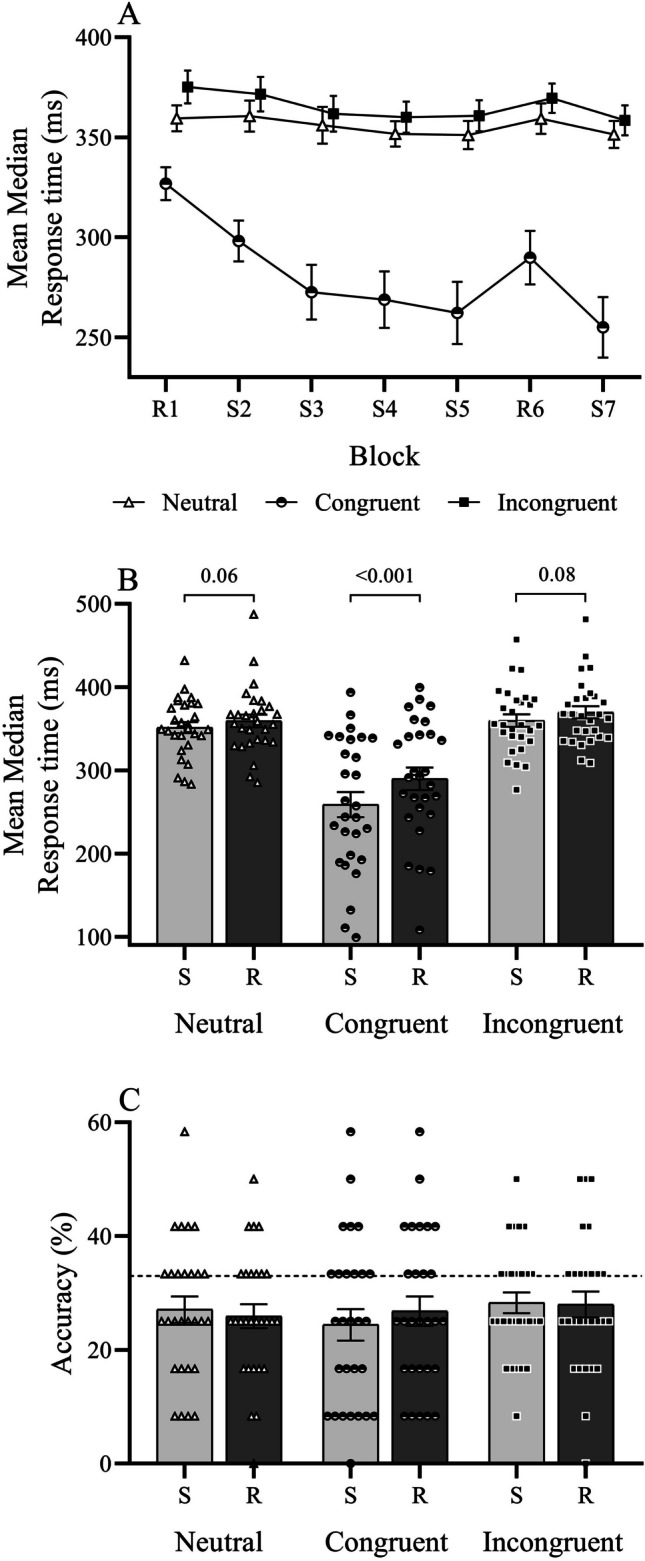
Mean median response times of a SRTT subliminally primed with neutral, congruent, or incongruent targets (**A**). Sequence specific learning evaluated over sequenced (S5,S7) and random (R6) blocks (**B**). Choice task accuracy among random and sequence blocks (**C**). Dotted line represents chance accuracy during the choice task (33%)

A significant prime main effect again illustrated the influence of subliminal response primes on response latency (*F*_1.2,32.5_ = 59.27, *p* < .001, η_p_^2^ = 0.69, β = 1.00; Fig. 3A), with responses under congruent (−81.1 ± 10.6 ms; *p* < .001), but not incongruent primes (9.27 ± 3.98 ms; *p* = .08), significantly faster than neutral. Sequence specific learning was observed (sequence main effect: *F*_1,27_ = 21.89, *p* < .001, η_p_^2^ = 0.45, β = 1.00) with response times of sequenced blocks (S5, S7) significantly faster than random blocks (R6) (−16.4 ± 3.5 ms; *p* < .001). Moreover, the degree of learning differed among the three prime conditions (prime-by-sequence interaction: *F*_1.6,43.9_ = 6.98, *p* = .004, η_p_^2^ = 0.21, β = 0.86). Response times were significantly faster during sequenced compared with random blocks under congruent primes (*t*_27_ = −4.6, *p* < .001, *d* = −0.87; one-tailed) and approached significance under neutral (*t*_27_ = −2.14, *p* = .06, *d* = −0.40) and incongruent primes (*t*_16_ = −2.02, *p* = .08, *d* = −0.38; Fig. 3B). Between conditions, the difference in response time between sequence and random blocks was significantly greater under congruent (*W*_28_ = 3.05, *p* = .002) but not incongruent primes (*W*_28_ = −0.02, *p* = .98) when compared with neutral.

#### Rate of performance gain

A multilevel comparison of response time change compared with baseline (R1) was not possible due to abnormally distributed data in blocks S3 through S5. However, compared with baseline, response times differed significantly by the first sequential block (S2: *F*_1.6,4.9_ = 11.48, *p* < .001, η_p_^2^ = 0.30, β = 0.98), with response times under congruent (−29.83 ± 7.55 ms, *p* = .002), but not incongruent primes (−4.64 ± 4.84 ms, *p* = 1.00), significantly faster than neutral.

#### Sequence and prime awareness

Choice task accuracy averaged 25–28% and was comparable among prime conditions and blocks (main and interactions effects *F* < 0.5). The difference between random and sequence task accuracy ranged from −2 to 3 % and was not significantly different, χ^2^_(2)_ = −0.58, *p* = .75, indicating that responses were, on average, driven by random selection. Furthermore, with increasing task practice and irrespective of prime condition, task accuracy remained unchanged over the course of the experiment, χ^2^_(2)_ < 0.60, *p* > .75. Exploratory associations between the degree of sequence specific learning and choice task accuracy showed no significant association among prime conditions (*p* > .1). Prime detection accuracy converged at 60.5 ± 0.9%, significantly below the intended 66% convergence rate, χ^2^_(2)_ = −4.0, *p* < .001, with an average of 2.5 ± 0.2 lines. Exploratory associations between the degree of sequence specific learning and individual prime detection thresholds showed no significant association among prime conditions (*p* > .9).

### Discussion

The capacity to select and inhibit movements at appropriate points in space and time underpins the success of temporally organised behaviour. Using the SRTT overlaid with subminimal masked primes, we examined if modulating response conflict during sequence learning differentially modulated implicit skill acquisition. We showed over two experiments that congruent primes shifted response latency and led to greater sequence specific learning compared with both neutral and incongruent primes, an effect that emerged independent of sequence awareness. Moreover, incongruent primes appeared to attenuate sequence learning (Experiment 1), particularly in the absence of sequence awareness.

#### Prime congruency effect on sequence specific learning

Sequence specific learning was observed under both neutral and congruent prime conditions (Fig. 2A, B, Fig. 3A, B), yet the magnitude was significantly greater with congruent primes, suggesting that in addition to baseline congruency effects, prime congruency may also mediate implicit learning. Implicit learning is driven by selectively attending task relevant stimuli (e.g., Baker et al., 2004; Jiang & Chun, 2001; Turk-Browne et al., 2005), whereas prime–target congruency is thought to facilitate perceptual processing by shifting attention to the target location (Scharlau, 2002). Environments that provide a framework to organise complex information can enhance the efficiency of visual processing (Biederman, 1972). Congruent subliminal stimuli presented prior to a target may have provided one such schema, allowing for the clearer identification of sequential regularities embedded in the task. Increased salience of sequential regularities may have elevated sequence awareness through boundaries of consciousness and altered the representational status of acquired knowledge (Esser & Haider, 2017; Esser et al., 2022), as illustrated by six participants who correctly reported sequence awareness with congruent primes in Experiment 1. Qualitative changes in task performance, including a steep change of response times (Rose et al., 2010), can indicate shifts of awareness during an implicit learning task. The profile of response time reduction with congruent primes (Fig. 2A) in addition to a handful of participants who correctly reported sequence awareness in this condition questions whether subliminal primes merely modified the representational status of sequential knowledge. To explore this further, we reexamined sequence specific learning among participants with zero sequence awareness and again observed a prime-congruency effect on learning (Fig. 3C). Visual observations indicate a gradual change in task performance without sequence awareness (Fig. 2B) as opposed to the rapid reduction of response times among individuals with sequence awareness (Fig. 2C). However, the profile of response time reduction noted in Experiment 2 does not align with this view, despite no awareness of a ten-digit sequence. Note that the sequence structure applied in Experiment 2 was chosen to reduce the development of sequence awareness. Despite the success of this measure, given that only four participant reported explicit sequence awareness, this sequence structure results in continuously alternating four-digit chunks. Note however, that pseudorandom sequences (random blocks) also followed the same chunk structure, which may explain the response time reduction between random Blocks 1 and 6 under congruent prime conditions.

The development of explicit sequential knowledge reduces reliance on stimulus processing (Haider et al., 2011; Koch, 2007). In line with this notion, participants in Experiment 1 that developed sequence awareness were unaffected by incongruent primes (Fig. 2C), whereas participants without sequence awareness displayed little to no sequence-specific learning (Fig. 2B, E). A lack of sequence-specific learning under incongruent primes suggests that response conflict and the need to reorient behaviour in the absence of sequence awareness hinders the ability to form cross-temporal contingencies (Schwarb & Schumacher, 2012). While our experimental design does not permit insight to the locus of implicit learning, the very notion of subliminal priming directs observed phenomena towards a stimulus or stimulus–response-based mechanism of learning in which attention, correctly or incorrectly, is guided towards stimuli. Upon task switching, selective inhibition permits flexible loading of new task sets, yet residual inhibition leads to downstream behavioural costs when reactivating a recently inhibited task (Mayr & Keele, 2000). Conflict, and the need to suppress irrelevant information during the stimulus–response transition, may evoke a similar nature of inhibitory tone that (1) weakens the association among stimulus–response compounds and (2) incurs behavioural costs when reactivating task associations.

Our observations broadly align with the effects of prime congruency—where congruent primes facilitate and incongruent primes inhibit learning—but they diverge from findings regarding the modulation of demands on controlled response selection or prepotent response inhibition. For instance, altered stimulus–response rules (Deroost & Soetens, 2006; Koch, 2007) and incongruent subliminal primes (Experiment 1) produced contrasting effects on learning. Notably, the explicitness of the response selection process differs significantly between these two conditions. Response remapping is believed to increase demands on controlled response selection, engaging higher-order cognitive processes that enhance information processing (Friedman & Robbins, 2022). In contrast, subliminal priming activates lower-level sensory and perceptual areas, such as the visual cortex, and operates outside of conscious awareness, preventing deliberate response evaluation or adjustment. When focusing on semantic stimulus properties by rendering spatial stimuli irrelevant, implicit learners were unaffected by behavioural inhibition, as indicated by the Simon effect between compatible and incompatible stimuli (Koch, 2007). In contrast, explicit learners demonstrated greater sequence-specific learning (Koch, 2007). These altered states of awareness and mechanisms of conflict induction suggest that the hierarchical nature of the stimulus–response process can profoundly influence the acquisition and production of sequential behaviour. While sequence awareness and the engagement of top-down processing in Experiment 1 largely mitigated prime-mediated response conflict (Fig. 2C, F), participants without sequence awareness, and presumably engaged in bottom-up stimulus processing, did not experience this effect (Fig. 2 B, E). Notably, this effect was not replicated in Experiment 2, suggesting that in addition to the muted sequence specific learning from reduced sequence exposure and a more complex sequence structure, a weaker conflicting stimulus (16.7 vs. 25 ms) does not sufficiently activate the neural representation of a conflicting stimulus.

#### Effect of prime congruency on response latency

A response latency shift under congruent prime conditions aligns with the notion that information processed beyond our awareness can inform upcoming action schemas and lead to the faster selection and release of motor programs (Eimer & Schlaghecken, 2002; Kilner et al., 2003). However, we did not observe an attenuation of response times with incongruent primes. Previous accounts of the prime congruency effect have been made using binary response tasks (i.e., left/right, yes/no; Seiss & Praamstra, 2004), yet our task permitted four possible release options. The maintenance of several release options requires the coordinated suppression of multiple responses upon target presentation. Irrespective of the response bias induced by incongruent primes, the magnitude of inhibitory drive necessary to suppress three responses under neutral prime conditions appears behaviourally comparable to that of the incongruent condition.

#### Sequence and prime awareness

An average of 2.5 (±0.2) lines were required in the mask to prevent conscious perception of the prime, significantly lower than the 20 lines used in the experimental conditions. Despite the success of this procedure, the small number of lines necessary to conceal the prime may explain the lack of effect observed under incongruent prime conditions in Experiment 2. Whereas congruent primes facilitated performance and learning—potentially by reinforcing neural representations—the weaker inhibitory stimulus of the incongruent primes may not have been sufficient to effectively conflict with the intended response (Dehaene et al., 1998). Sequence awareness, assessed by the percentage of correct responses in the choice task for both random and sequenced blocks, showed accuracy at chance level, confirming that participants were unaware of the underlying sequences. This suggests that prime-mediated learning influenced lower-level processing. However, unlike Haider et al.’s (2011) wagering task, the choice task here included only ten trials per block (one for each sequence element), which may not have reliably captured sequence awareness due to high response variability.

## Limitations

The findings of these experiments should be considered in light of several limitations. Although common practice to evaluate sequence awareness through questionnaire (Hama & Leow, 2010), we were unable to directly examine sequence awareness after each condition and therefore unable to determine the development of explicit sequence knowledge over the course of the experiment. Despite being statistically well-powered, care should be taken with the interpretation of prime-mediated learning in Experiment 1 due to the high attrition rate, which may lead to an overestimation of power in small samples. Modifying the sequence structure in Experiment 2 largely mitigated this issue. However, it is important to characterise additional factors that significantly affect behavioural performance during experimental sessions, such as sleepiness (Hoddes et al., 1973), attention, and fatigue. Although we demonstrated a divergent effect of prime congruency on sequence specific learning, the degree to which this effect represents a true modification to learning and not adaptation to prime stimuli remains unclear. Appending each SRTT with an evaluation of sequence specific learning under neutral prime conditions would allow inference of learnt associations in the absence of primes. Despite confirming a prime mediated effect on response latency, baseline response latency between each condition was not evaluated, and thus the contribution of general motor adaptation to the prime mediated learning effect cannot be established. Lastly, the degree to which higher-order response control mechanisms contributed to prime mediated learning is unknown. While associations between lower-level mechanisms of response control (corticospinal excitability) and sequence specific learning was inconclusive (see Supplement One), the inclusion of established response control paradigms, such as the stop signal and flanker tasks, would allow for a hierarchical evaluation of response control during implicit sequence learning.

## Conclusions

Overlaying the SRTT with subliminal masked primes allowed us to differentially weigh response tendencies during the response encoding and preparation phase of implicit motor sequence learning. We show over two experiments that congruent primes may accentuate learning by increasing the salience of stimulus–response compounds, but at the cost of increased awareness. On the other hand, incongruent primes may to attenuate performance, suggesting that the induction of response inhibition during stimulus encoding or response selection may prevent the formation of cross-temporal contingencies necessary for incidental skill acquisition.

## Supplementary information

Below is the link to the electronic supplementary material.

Supplementary file1 (DOCX 499 KB)

## Data Availability

Data that support the findings of this study can be found on Science Data Bank [10.57760/sciencedb.12986].

## Code availability

Code that supports the findings of this study can be found on Science Data Bank [10.57760/sciencedb.12986].
