# Supplementary material for: Subliminal priming modulates motor sequence learning
Source: Mem Cognit. 2024 Nov 21;53(5):1539–50. doi: 10.3758/s13421-024-01668-8 (PMC12307551; doi:10.3758/s13421-024-01668-8)
Supplement: Supplementary file 1 — Supplementary file1 (DOCX 499 KB) [file 13421_2024_1668_MOESM1_ESM.docx]

**Supplement one – Transcranial Magnetic Stimulation Corticospinal Excitability**

**Introduction**

Sequential behaviour demands that movements are selected and inhibited at appropriate points in space and time, a process mediated across hierarchical mechanisms of cognitive and motor control (Beck & Hallett, 2011). Response priming, the provision of a stimulus prior to a response, is believed to activate neural representations of the primed stimulus. For instance, priming left or right responses in a Flanker task can generate preparatory activity in the corresponding motor cortex (Seiss & Praamstra, 2004). In cases of incongruent priming, this preparatory activity must be suppressed before the correct motor response can be executed (Seiss & Praamstra, 2004). The controlled release of a motor response depends on the sensitivity of corticospinal circuits which mediate the flow of motor commands (Floeter & Rothwell, 1999). Excitatory and inhibitory interactions ensure that the appropriate pattern of activation descends the spinal cord—a phenomenon measurable with transcranial magnetic stimulation (TMS). Under conditions requiring reactive inhibition, corticospinal excitability (CSE) sharply reduces to prevent the execution of an unintended motor response (Coxon et al., 2006; Picazio et al., 2014). Moreover, dynamic changes in CSE reveal use-dependent changes in the functional architecture associated with motor learning (Pascual-Leone et al., 1994; Smyth et al., 2010). Given the association between CSE, behavioural control, and learning, the capacity to regulate CSE in light of altered stimulus congruency may determine the degree to which stimulus/response compounds are bound. Traditional measures of behavioural inhibition, such as the stop-signal task and go/no-go task, involve top-down cognitive control, engaging higher-order brain regions to generate an inhibitory response. Investigating the relationship between baseline CSE and primed sequence-specific learning may offer a new perspective into the lower-level physiological mechanisms underlying implicit response control during implicit sequence learning.

**Methods**

*Experimental procedure*

Participants attended one experimental session in which baseline CSE was measured before performing the serial reaction time task (see main text for details). Biphasic single- and paired-pulse TMS was delivered using a MagVenture X100 + Option TMS device and a Cool-B70 figure-of-eight coil (MagVenture, Denmark). Single-use surface electrodes (Ag/AgCl) were attached in a belly-tendon montage over the right first dorsal interosseous muscle (FDI) with the ground electrode placed over the ulna notch. The FDI motor hotspot was identified by placing the TMS coil tangentially on the scalp with the handle 45° to the sagittal midline over C3 (international 10-20 system, Herwig et al., 2003). Single pulses were delivered at 65% of maximum stimulator output (MSO) and the coil moved in 1 cm steps (lateral/vertical) until large and consistent motor evoked potentials (MEP) were identified. Resting motor threshold (rMT) was established by delivering 10 pulses to the motor hotspot at 35% of MSO and increased in 5% steps until 5/10 MEP’s exceeded 50 µV in peak-to-peak amplitude. MSO was then decreased in 1 % steps until < 5/10 MEPs reached 50 µV. One percent above this value was then registered as rMT. MEP amplitude, short-intracortical inhibition (SICI), and short-intracortical facilitation (SICF) was then pseudo-randomly measured. Ten single-pulses were delivered at 120% of rMT (MEP), ten paired-pulses with an inter-pulse interval of 2 ms at 80% and 120% of rMT (SICI), and ten paired-pulses with an inter-pulse interval of 2 ms at 120% and 90% of rMT (SICF).

*Data processing and analysis*

Electrophysiological data were imported into Matlab and averaged over MEP, SICI, and SICF conditions. Peak-to-peak amplitudes were calculated with SICI and SICF measures expressed as the ratio of unconditioned to conditioned MEP responses. Associations between TMS derived CSE and sequence specific learning were explored using Pearson’s or Spearman’s Rho correlation coefficient. Significance values corrected for multiple comparison using Bonferroni.

**Results**

Excitability data from three participants were discarded due to unstable FDI hotspots. Data from 21 participants were carried forward for analysis. Among all individuals, irrespective of sequence awareness or recall, only resting motor threshold displayed a significant negative association with the degree of sequence specific learning under neutral prime conditions (Spearman’s rho; r=-0.52, p=0.046). No other significant or trend associations were observed (see Supplementary Figure 1). Among individuals without sequence recall (14) or without sequence awareness (8), no significant or trend associations were observed (p > 0.16 and p > 0.35, respectively).

**Discussion**

Incongruent primes are known to evoke preparatory activity in the primed motor cortex which is later suppressed before selecting the correct response (Seiss & Praamstra, 2004). Given the association between CSE and both behavioural inhibition (Picazio et al., 2014) and motor learning (Pascual-Leone et al., 1994; Smyth et al., 2010), the capacity to regulate the sensitivity of the motor cortex to depolarisation may dictate the influence of response conflict to the binding of stimulus/response pairs. Despite trend associations, which did not persist upon the removal of participants with sequence awareness and recall, baseline CSE did not associate with sequence specific learning under any prime condition. A lack of association is likely due to the static nature of baseline CSE measurements. Given the change of CSE that can accompany motor learning (Pascual-Leone et al., 1994; Smyth et al., 2010), profiling the change of excitability in relation to prime modulated sequence specific learning may better reflect the role of CSE in conflict mediated learning. Moreover, SICI and SICF are magnitude-based measures of excitability (Paci et al., 2021). Given the prime-target interval (100ms) and the postulated residual inhibition that may persist among stimulus-response compounds, temporal measures of cortical inhibition, such as the cortical silent period, may better capture the inhibitory processes underling conflict mediated learning. Beyond the motor cortex, the influence of subliminal masked primes is likely processed among conflict resolution networks that run loops through the anterior cingulate cortex (conflict monitoring) and lateral prefrontal cortex (control adaptation) (Egner & Hirsch, 2005; Kerns et al., 2004). Contrasting the engagement of these nodes during the SRTT under opposing degrees of conflict would permit insight to the neural substrates of conflict mediated learning.

*Limitations*

Given the exploratory nature of excitability measures in this study, CSE was measured before the first respective condition so as not to impact the timing between experimental conditions. However, CSE is known to change over the course of an experiment (Pascual-Leone et al., 1995). Static baseline measures may not accurately capture the dynamic role CSE plays in response control during sequence learning. Furthermore, while CSE was measured as an indicator lower-level response control, the inclusion of established response control paradigms, such as the stop signal task and flanker task, may allow a hierarchical evaluation of response control during implicit sequence learning.

**References**

Beck, S., & Hallett, M. (2011). Surround inhibition in the motor system. *Experimental Brain Research*, *210*(2), 165–172. https://doi.org/10.1007/s00221-011-2610-6

Coxon, J. P., Stinear, C. M., & Byblow, W. D. (2006). Intracortical Inhibition During Volitional Inhibition of Prepared Action. *Journal of Neurophysiology*, *95*(6), 3371–3383. https://doi.org/10.1152/jn.01334.2005

Egner, T., & Hirsch, J. (2005). Cognitive control mechanisms resolve conflict through cortical amplification of task-relevant information. *Nature Neuroscience*, *8*(12), 1784–1790. https://doi.org/10.1038/nn1594

Herwig, U., Satrapi, P., & Schönfeldt-Lecuona, C. (2003). Using the International 10-20 EEG System for Positioning of Transcranial Magnetic Stimulation. *Brain Topography*, *16*(2), 95–99. https://doi.org/10.1023/B:BRAT.0000006333.93597.9d

Kerns, J. G., Cohen, J. D., MacDonald, A. W., Cho, R. Y., Stenger, V. A., & Carter, C. S. (2004). Anterior Cingulate Conflict Monitoring and Adjustments in Control. *Science*, *303*(5660), 1023–1026. https://doi.org/10.1126/science.1089910

Paci, M., Di Cosmo, G., Perrucci, M. G., Ferri, F., & Costantini, M. (2021). Cortical silent period reflects individual differences in action stopping performance. *Scientific Reports*, *11*(1), 15158. https://doi.org/10.1038/s41598-021-94494-w

Pascual-Leone, A., Grafman, J., & Hallett, M. (1994). Modulation of Cortical Motor Output Maps During Development of Implicit and Explicit Knowledge. *Science*, *263*(5151), 1287–1289. https://doi.org/10.1126/science.8122113

Pascual-Leone, A., Nguyet, D., Cohen, L. G., Brasil-Neto, J. P., Cammarota, A., & Hallett, M. (1995). Modulation of muscle responses evoked by transcranial magnetic stimulation during the acquisition of new fine motor skills. *Journal of Neurophysiology*, *74*(3), 1037–1045. https://doi.org/10.1152/jn.1995.74.3.1037

Picazio, S., Veniero, D., Ponzo, V., Caltagirone, C., Gross, J., Thut, G., & Koch, G. (2014). Prefrontal Control over Motor Cortex Cycles at Beta Frequency during Movement Inhibition. *Current Biology*, *24*(24), 2940–2945. https://doi.org/10.1016/j.cub.2014.10.043

Seiss, E., & Praamstra, P. (2004). The basal ganglia and inhibitory mechanisms in response selection: evidence from subliminal priming of motor responses in Parkinson’s disease. *Brain*, *127*(2), 330–339. https://doi.org/10.1093/brain/awh043

Smyth, C., Summers, J. J., & Garry, M. I. (2010). Differences in motor learning success are associated with differences in M1 excitability. *Human Movement Science*, *29*(5), 618–630. https://doi.org/10.1016/j.humov.2010.02.006


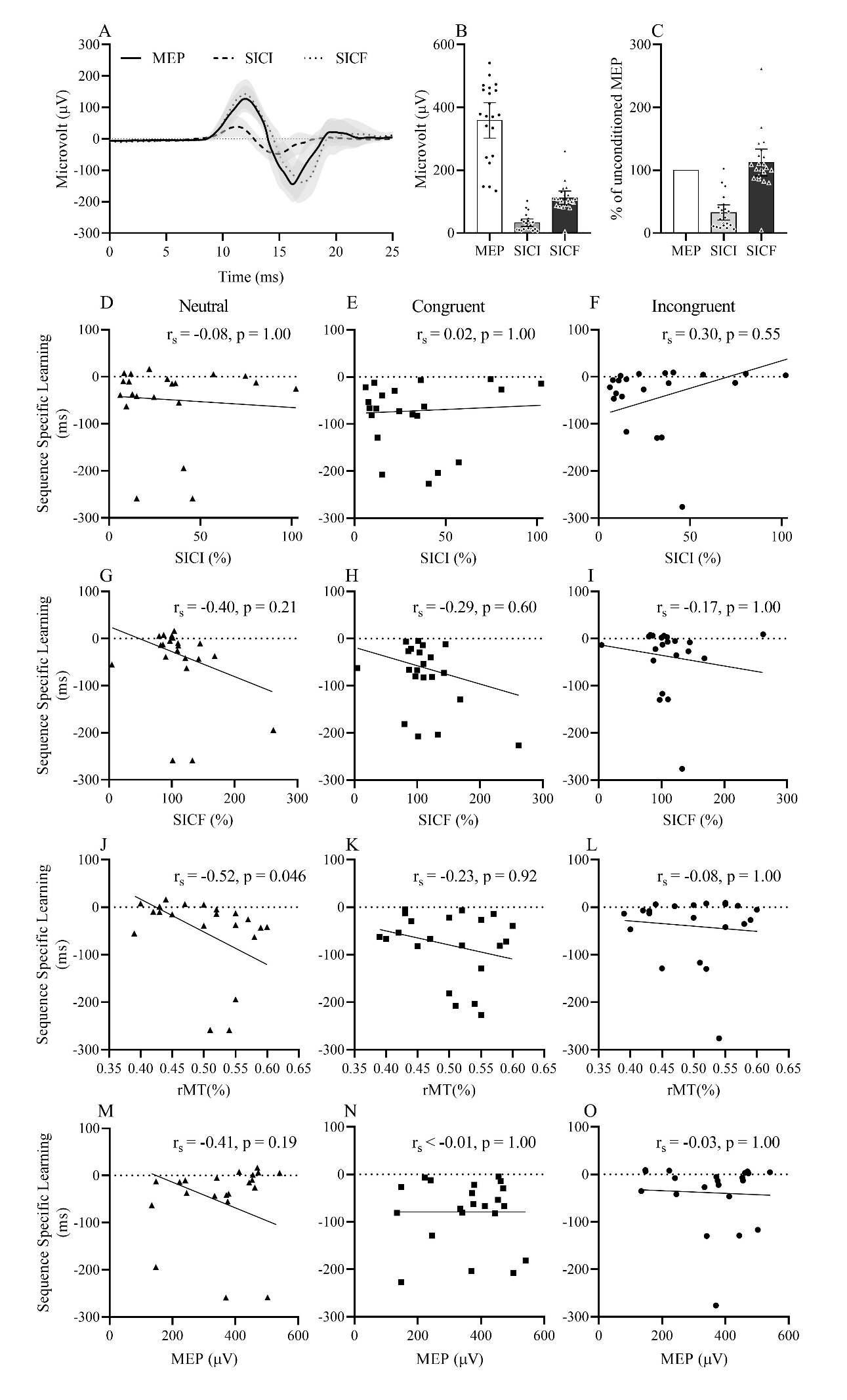
Supplementary Figure 1. Motor evoked potential (MEP), short intracortical inhibition (SICI), and short intracortical facilitation (SICF) traces (A), with absolute (B) and relative (C) values. Panels D–F show the association between SICI and sequence-specific learning under neutral, congruent, and incongruent subliminal prime conditions in the serial reaction time task. Panels G–I display the same associations for SICF, J–L for resting motor threshold (rMT), and M–O for MEP.
